# High-Throughput Robotic GIWAXS at ALS SAXS/WAXS Beamline

**DOI:** 10.1021/photonsci.5c00017

**Published:** 2025-10-22

**Authors:** Eric Schaible, Ivan Galikeev, Matthew Roizin-Prior, Garret Birkel, Yunfei Wang, Wiebke Koepp, Raja Vyshnavi Sriramoju, Harold Barnard, Chinweike Osubor, Camille Molsick-Gibson, Piotr Gach, Sujoy Roy, Xiaodan Gu, Dylan McReynolds, Alexander Hexemer, Lucas Kistulentz, Damon English, Dilworth Y. Parkinson, Chenhui Zhu

**Affiliations:** † Advanced Light Source, 1666 Lawrence Berkeley National Laboratory, Berkeley, California 94720, United States; ‡ School of Polymer Science and Engineering, 5104 University of Southern Mississippi, Hattiesburg, Mississippi 39406, United States

**Keywords:** advanced light source (ALS), SAXS/WAXS, GIWAXS, grazing-incidence wide-angle X-ray scattering, high-throughput, thin films, grazing incidence, robotic sample changer, web-based sample tracking system, automation

## Abstract

We present the development of a high-throughput, robotic GIWAXS platform for automated thin-film characterization at the ALS SAXS/WAXS beamline. The system features a UR5e robotic arm, a helium-purged sample chamber, integrated beamline data acquisition routines, and a web-based interface for sample tracking. Designed for seamless compatibility with computational workflows, the control software enables real-time data transfer, analysis, and autonomous experimentation, greatly increasing throughput with additional gains in noise reduction and resource management. The platform also supports future integration with synthesis and sample-processing systems, such as Sciprios and North Robotics, paving the way for closed-loop, data-driven materials research and establishing a means to accelerate the pace of materials innovation.

## Graphical abstract

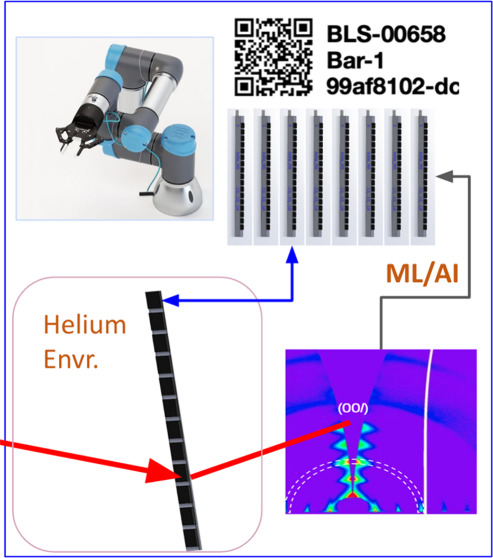

## 1 Introduction

Self-driving laboratories—also referred to as autonomous or closed-loop experimental platforms—are increasingly recognized as transformative tools in materials research.^1–7^ By integrating robotics, machine learning, and high-throughput characterization, these systems enable fully automated workflows that span materials synthesis, processing, real-time or in situ characterizations, and adaptive decision-making guided by artificial intelligence (AI). In complex domains such as polymer science, nanocrystals, and halide perovskite materials, where optimal performance depends on navigating high-dimensional, nonlinear parameter spaces, autonomous experimentation offers the potential to dramatically accelerate discovery, reduce human error, and improve reproducibility.

Recent advances have demonstrated the feasibility of autonomous experimentation across a variety of large-scale research facilities.^8^ Notable implementations include autonomous workflows at neutron beamlines,^9^ synchrotron beamlines,^10–13^ and nanoscience centers.^5,14–16^ Concurrently, machine learning techniques are being increasingly tailored to support such workflows, particularly in synchrotron beamline environments.^17^

Robotic systems have played a central role in integrating sample processing and characterization into autonomous experimental pipelines.^18^ They are also widely used for automated sample exchange at various neutron and X-ray scattering instruments. Examples include Bio-SANS^19^ and the Liquids Reflectometer^20^ at Oak Ridge National Laboratory; BioSAXS (SIBYLS) at the Advanced Light Source (ALS);^21^ FMX at the National Synchrotron Light Source II (NSLS-II);^22^ GI-SAXS/WAXS at the Complex Materials Scattering (CMS) beamline at NSLS-II; and XRD at Stanford Synchrotron Radiation Lightsource.^23^ A robotic pendant-drop system has also been developed for containerless liquid handling in X-ray photon correlation spectroscopy (XPCS) experiments at the Advanced Photon Source.^24^

Synchrotron-based grazing-incidence (GI) wide-angle X-ray scattering (GIWAXS) is a critical technique for probing the structural, morphological, and phase behavior of thin films and nanostructured materials.^25–27^ It offers valuable insights into crystallinity, polymorphism, and molecular orientation under diverse processing conditions—properties that directly affect the performance of functional materials such as organic semiconductors, block copolymers, and hybrid perovskites. At ALS, as well as other U.S. Department of Energy (DOE) user facilities, GIWAXS remains in high demand and continues to support numerous high-impact studies.

At the ALS SAXS/WAXS beamline, we have implemented some automated sample alignment and data acquisition routines, along with multi-sample loading strategies (e.g., bar-mounted samples) to streamline the measurement process. However, realizing continuous and truly autonomous experimentation—particularly in anticipation of increased beam flux and integration with advanced computational tools following the ALS Upgrade—will require robust, reliable robotic sample exchange systems. This need is especially timely, as the SAXS/WAXS capabilities are being relocated from beamline 7.3.3 to beamline 4.3.1 as part of the upgrade.

In this work, we present the design and implementation of a high-throughput robotic GIWAXS measurement system. The platform integrates a collaborative robotic arm (UR5e) for fully automated sample handling and exchange, a centralized database for sample tracking, and automated GIWAXS acquisition protocols—all coordinated through a unified software interface. This architecture significantly improves measurement throughput and operational efficiency, enabling robust, unattended data collection suitable for integration into autonomous experimental workflows. We note that the essential components developed here are largely compatible with robotic GISAXS as well as robotic transmission SAXS/WAXS measurements planned for the near future.

While the realization of fully self-driving laboratories remains a long-term objective, this robotic GIWAXS platform provides a crucial technological foundation for intelligent, high-throughput structural characterization. It serves as an enabling step toward future AI-guided materials discovery workflows at synchrotron beamlines. Detailed descriptions of the platform are provided in the following sections.

## 2 Previous Helium Box GIWAXS Geometry

The previous experimental geometry is described in detail in the Supporting Information (SI). Briefly, the incident X-ray beam passed through a copper collimation tube—which served as a pinhole to clean the beam—and impinged on a thin-film sample deposited on a silicon wafer under GI conditions. Scattered X-rays exit the plastic sample chamber—commonly referred to as the “helium box”—through a thin Kapton window and were detected by a Dectris Pilatus 2M detector downstream in ambient air. A beam stop equipped with a diode was placed just outside the Kapton window to block directly scattered X-rays from the window itself (Figure S1c) and to monitor the transmitted beam intensity. Both the pinhole and beam stop were manually fine-tuned during initial alignment. To reduce background from air scattering, the chamber was continuously purged with helium gas, maintaining an oxygen concentration below 0.25%. Unlike vacuum environments, a helium environment is compatible with various in situ sample processing techniques, and thus enables a wider range of testing capabilities at the beamline.

Despite its merits, a major bottleneck in the previous experimental procedure was the helium purging process: reducing ambient oxygen concentration from ∼20% to below 0.25% typically required approximately 35 min of continuous helium flow. This delay was primarily due to components of the box that were difficult to seal airtight, such as the side window used for manual sample loading (Figure S1, marked by arrows). Another significant constraint was the manual sample exchange process. Sample bars had to be loaded and unloaded through the same side window of the helium box by on-site personnel, regardless of the time of day. The loading process was further encumbered by the setup’s fragile design, which was often misaligned in the bar exchange process, further increasing testing time. This process imposed operational limitations—restricting after-hours data collection—and hindered automation, thereby limiting the system’s potential for high-throughput measurements.

## 3 Robotic GIWAXS System

To enable full workflow automation, we redesigned several critical components, including a new helium chamber. A web-based interface—integrated with the ALS proposal system—was developed to allow users to enter sample and measurement details and generate QR codes for labeling individual sample bars. An inverted UR5e collaborative robot is mounted above the helium box to enable automated sample handling. A new control software interface was also implemented to coordinate robotic movements, QR code scanning, gate valve operation, sample alignment, and data acquisition—establishing the foundation for closed-loop, autonomous experimentation tailored to long-term experimental goals and made both modular and adaptable to future needs. A detailed description of the redesigned components is provided below.

### 3.1 New Helium Sample Chamber

To enable fully robotic operation of GIWAXS, the sample chamber has been redesigned, as shown in Figure 1. The new helium chamber consists of two main components: an aluminum base plate and an acrylic top. The aluminum base plate incorporates two bespoke electrical feedthrough panels that accommodate both standard and custom connectors, thereby facilitating system integration and future upgrades. The acrylic top includes an access door for convenient maintenance on one side and a computer-controlled, pneumatically actuated gate valve on the other, replacing the previously manual window seal to support robotic operation. Kapton windows are used to seal the entrance and exit of the chamber while allowing X-ray transmission. The top encloses the entire chamber, with the base plate serving as its foundation.

**Figure 1:**
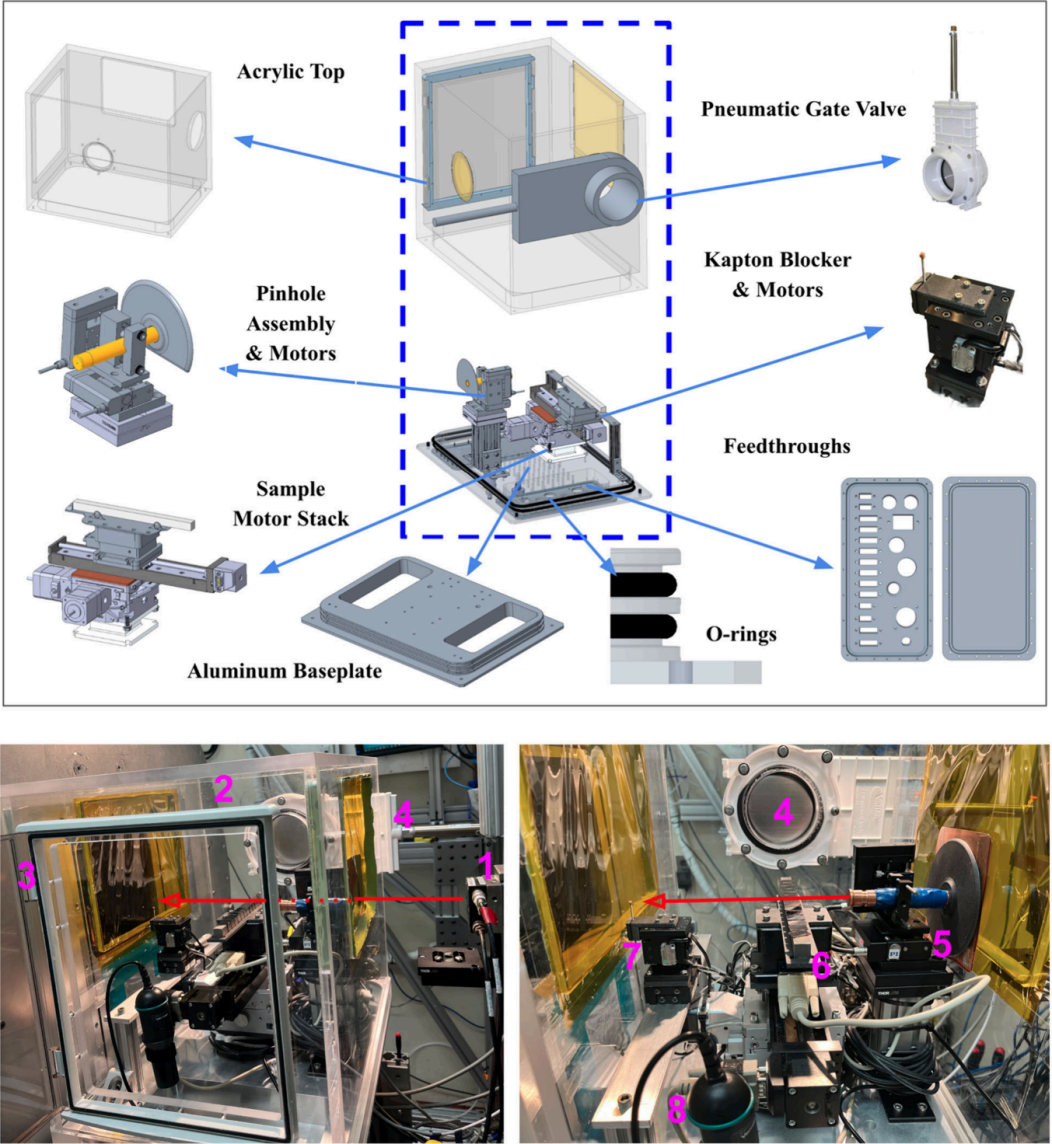
(Top) Key components of the new helium chamber setup shown in CAD. (Bottom) The setup as implemented at the beamline: (1) end of the synchrotron beam pipe, where X-rays come out, (2) helium chamber, (3) access door, (4) gate valve, (5) pinhole assembly and motor stack, (6) a bar of samples, (7) Kapton blocker and motor stack, (8) Vernier oxygen sensor.

The acrylic top of the helium chamber is sealed to the baseplate using O-ring piston seals and can be easily retracted to allow access for modifications or maintenance inside the chamber. The new chamber design was developed to provide a better seal with fewer potential leakage points. Helium flow is controlled by a solenoid valve (on/off) and regulated by a proportional valve operating within a feedback loop. A Vernier oxygen sensor provides real-time measurements to a LabVIEW control system, which adjusts the helium flow to displace oxygen until a specified set point is reached. Once the target oxygen concentration is achieved, a PID control loop maintains this level. Using this new setup, the oxygen concentration can be reduced to below 0.25% within 10 min (Figure S2), a significant improvement over the approximately 35 min required in the previous setup. To maintain safe operating conditions, the net pressure difference between the chamber interior and the external atmosphere is limited to below 0.5 psi. This is achieved using a check valve as the exit flow path, which automatically opens if the pressure exceeds this threshold, thereby ensuring consistent and safe purging behavior.

The pinhole assembly (Figure 1) is now mounted on a motorized X/Y stage inside the helium chamber, allowing precise alignment from outside the hutch while the X-ray beam is active. Beam positioning is monitored using a camera in combination with a 45° mirror, which is inserted into the beam pipe approximately 10 cm upstream of its end (SI) and positioned slightly above the X-ray beam to provide a clear view along the pinhole and mark the beam position relative to it. To minimize unwanted scattering, a large copper square (approximately 8 cm across) blocks potential wide-angle scattering from the mica window at the end of the synchrotron beam pipe while a copper tube (approximately 8 mm in diameter) with a small pinhole at its end further suppresses scattering from the Kapton entrance window of the chamber.

The beam stop assembly has also been redesigned to include a Kapton blocker (Figures 1 and S1) located inside the helium enclosure, positioned just upstream of the Kapton exit window, and a diode (in a separate beam stop) placed directly in front of the Pilatus 2M detector (Figure S3). During GI alignment, the Kapton blocker retracts from the beam path allowing post-sample beam intensity measurement, then returns to block the direct beam during GIWAXS data collection. This design significantly reduces background, resulting in improved GIWAXS image quality (Figures 2, S4, and S5).

**Figure 2:**
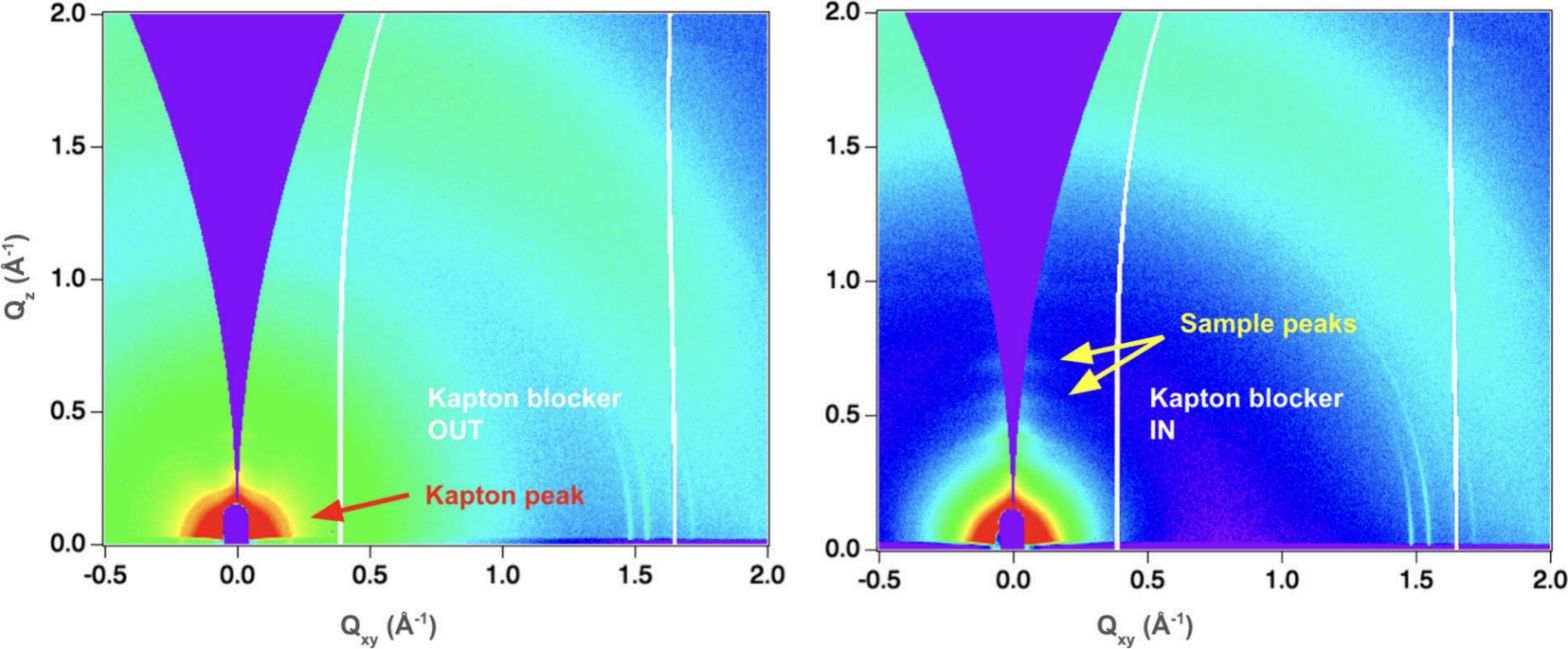
GIWAXS data quality comparison, GIWAXS of a weak scattering sample with the Kapton blocker out (left) and in (right). Note that when the Kapton blocker is out, there is a clear Kapton peak in the lower q area, besides a higher background scattering. Weak sample peaks of interest become more visible (marked by yellow arrows) when the Kapton blocker is in (right). GIWAXS data has been converted from raw detector images into the reciprocal *Q*_*z*_–*Q*_*xy*_ space.

### 3.2 Sample Tracking Interface

Users will prepare thin-film samples on wafer substrates, which are then mounted onto aluminum bars that can be ordered directly from vendors such as McMaster-Carr (see more in SI) Initially, samples will be positioned at fixed, regular intervals—e.g., 12 mm apart—to simplify automated sample switching (Figure S6). Some space on either end of the bars will be reserved for easy bar handling, packaging, and shipping.

To streamline information transfer, a web-based interface (Figure 3A) has been developed, connected to a SciCat (Scientific Catalog)^28^ backend via API calls, and integrated with the ALS proposal system (ALS hub). Users can log in using ORCID (Open Researcher and Contributor ID) accounts, select an existing general user or rapid proposal for which they have access, and input details such as bar, sample, and measurement parameters, including incident angles, number of spots per sample, and exposure time preferences.

**Figure 3:**
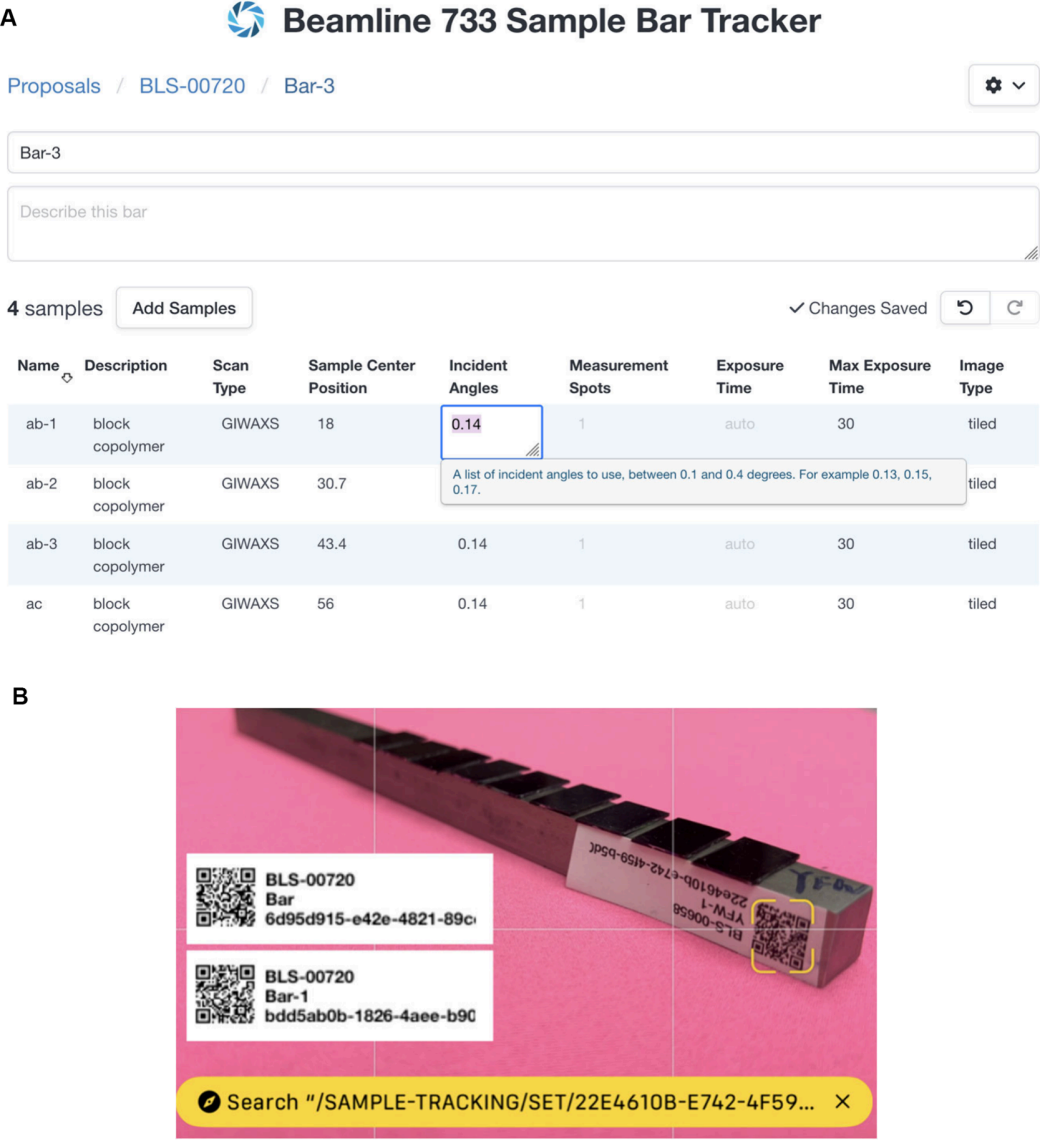
(A) An example screen from the sample tracking interface. After selecting a proposal ID and a bar, an editable table of samples appears. Here the incident angles list for sample ab-1 is being edited. (B) Printable QR codes used for automated sample identification.

The main portion of the interface consists of a sortable, editable table, showing columns for scan parameter information. The set of columns displayed is based on which “scan type” the user sets for each sample. (Currently only one “scan type” is defined but this feature will be leveraged in the near future.) While the user is editing, each table cell shows a brief description of the parameter, and automatically validates the entered value. All edits are synchronized with the back end automatically, and the user can “undo” and “redo” all their edits with the standard keyboard shortcuts. “Copy” and “paste” are also supported, and users can mass-select regions of the table to perform bulk edits. The overall effect is of a standard feature-complete spreadsheet that has been enhanced with scanning-specific features, making it easy to input data.

As a user describes their bars and samples, the application generates unique identifiers for each. These identifiers are stored alongside the user information. When the bars and samples are complete, the user can proceed to an interface for printing labels to place on the bars. Each label contains human-readable information, as well as a machine-readable QR code (Figure 3B).

In addition to the unique identifier, QR codes may encode fundamental metadata or URLs linking directly to shared documents (e.g., Google Sheets) containing relevant sample and measurement information. These QR codes are printed and attached to the bars, each one serving as a means for automatic sample and bar recognition to ensure seamless integration with the central software interface. When scanning is complete, this metadata will be stored alongside the resulting X-ray datasets, ensuring traceability and reproducibility. This approach eliminates the need for email exchanges or manual uploads.

This integrated sample handling, identification, and metadata management approach underpins fully automated, high-throughput characterization workflows, significantly enhancing efficiency, traceability, and reproducibility at synchrotron facilities.

### 3.3 UR5e Robotic Arm, Movement, and QR Code Scanning

The UR5e robotic arm (Universal Robots, obtained via Olympus Controls) was selected as the automated sample changer due to its intuitive operation and built-in safety features. To optimize workspace and accessibility, the UR5e is mounted in an inverted orientation above the helium chamber and adjacent to the sample tray table, as illustrated in Figure 4. The tray table, which remains folded against the wall when not in use, can be deployed to accommodate dozens of sample bars. Robotic movements are precisely synchronized with the beamline’s sample stages, data acquisition system, and helium chamber operations through the integrated control interface, ensuring coordinated and reliable performance throughout the automated workflow (Figure 5).

**Figure 4:**
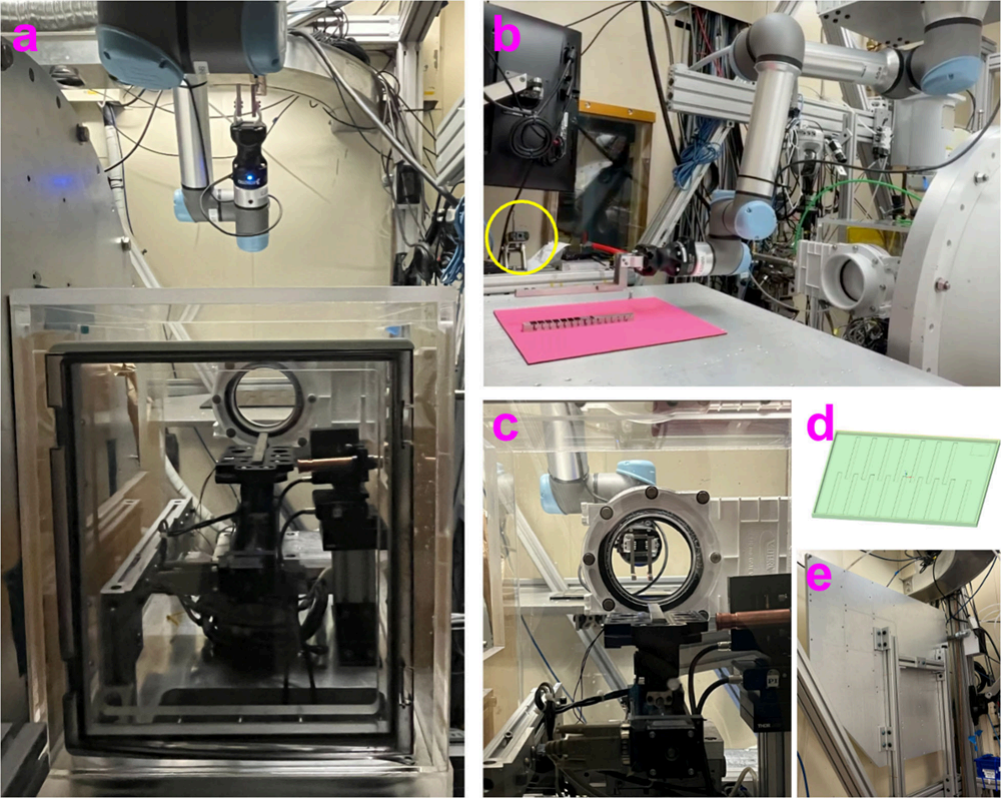
(a) UR5e robot positioned above the helium enclosure; (b) extended UR5e arm retrieving a sample bar from the sample tray (the QR code scanner is indicated by the yellow circle); (c) UR5e bar gripper reaching through the gate valve to access a sample bar inside the helium enclosure; (d) design of the sample tray; (e) sample tray table folded against the hutch wall when not in use.

**Figure 5:**
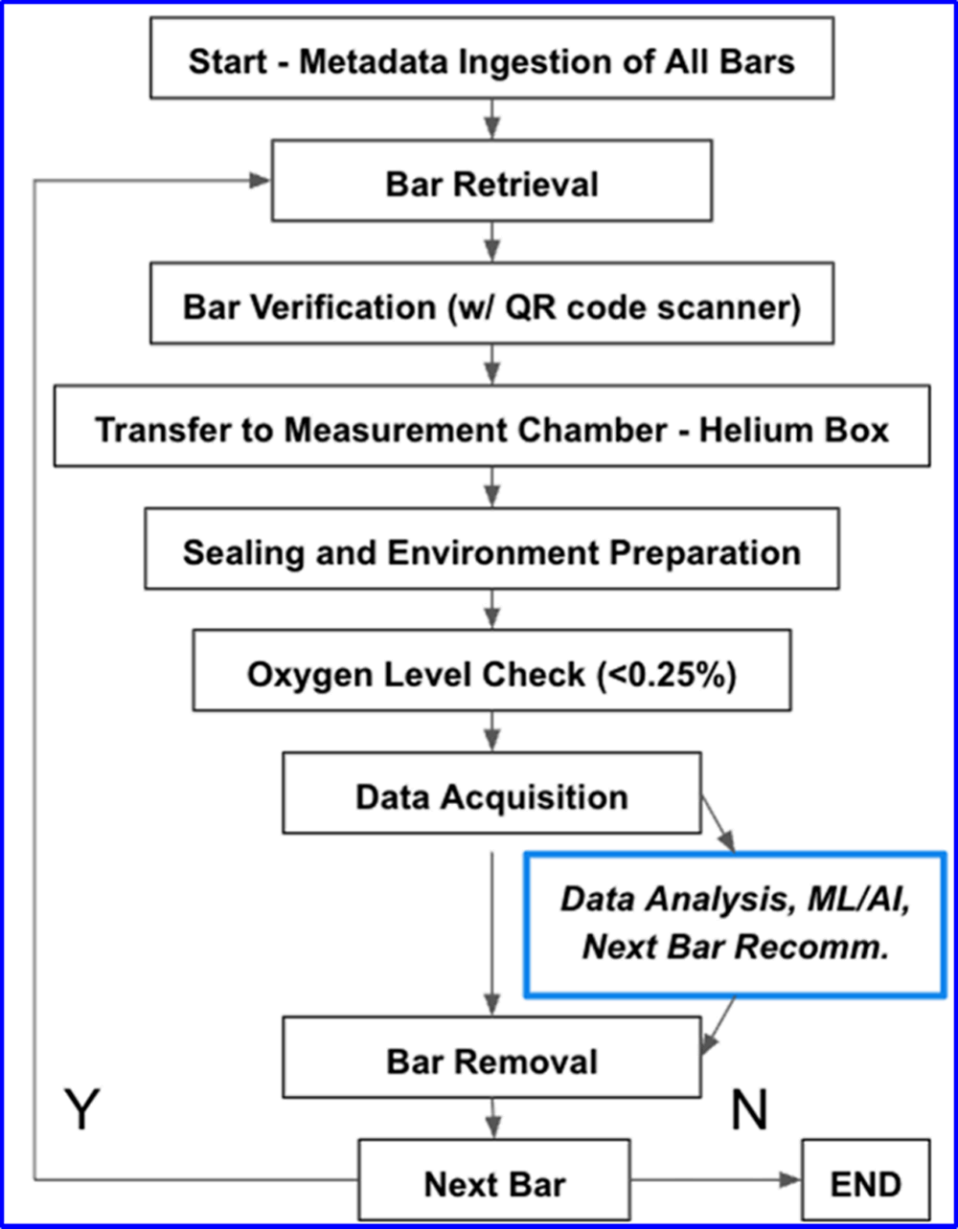
Basic work flow of high throughput GIWAXS of bar-type of samples. On-the-fly data analysis, ML/AI, next bar recommendations, and other advanced features are optional.

Following the initial robot setup, the automated workflow proceeds through a series of coordinated steps designed to enable high-throughput, unattended sample processing. In the default configuration, sample bar positions on the tray are predefined and fixed. The number of bars present is identified through image recognition using a Robotiq wrist-mounted camera on the UR5e robotic arm. This visual input informs the system’s internal map for locating and processing each bar.

The workflow begins with metadata ingestion: the robot scans the QR code on each bar to retrieve the associated bar, sample, and measurement information — previously entered by the user — via the SciCat API. To track experimental conditions, the Robotiq wrist camera periodically captures images of the tray to verify the bar positions.

By default, the robot processes all bars in sequence. However, users can encode priority metadata to allow selected bars to be processed first. This option is especially useful when beam time is limited and not all samples can be measured during a single run.

Immediately before measurement, each bar is retrieved from the tray, scanned again to confirm its identity, and transferred into the helium sample chamber. This transfer is synchronized with environmental preparation steps: the gate valve closes, and helium gas flow purges the chamber to an oxygen level below 0.25%.

During data acquisition, samples are aligned using the standard GI alignment procedure (see more detail in the next section, Figure 6). Cameras inside the helium chamber capture images of each sample location on the bar. These images are stored with the corresponding metadata and play a critical role in quality control. By comparing captured images with pre-uploaded sample maps, the system can detect issues such as misplaced, missing, or mixed-up samples due to shipping or handling errors.

**Figure 6:**
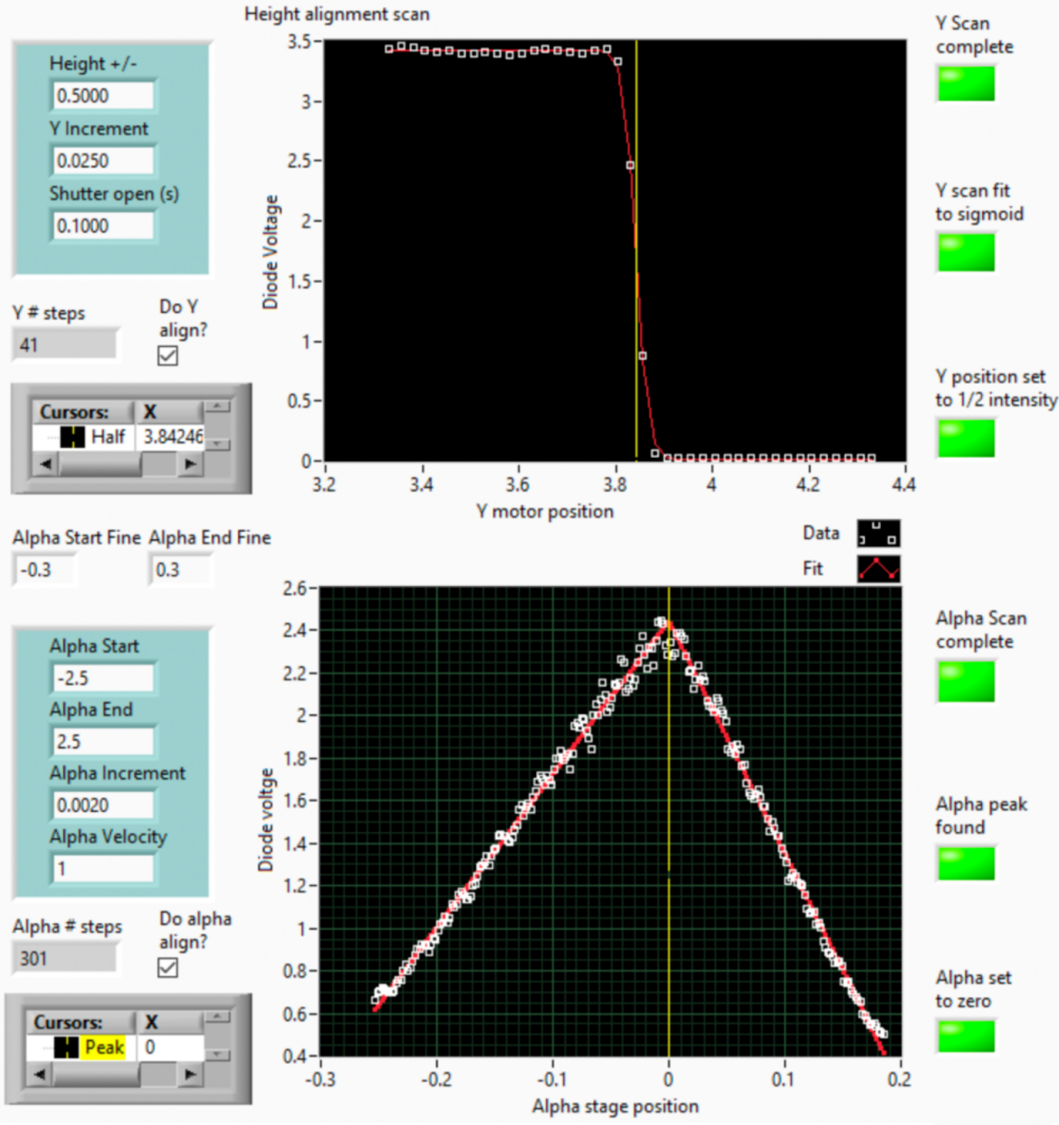
GI-alignment LabView routine for sample height (top) and incident angle (bottom). The vertical axis—diode voltage—represents the transmitted beam intensity after passing through the sample. In the top panel, the routine adjusts the sample height to allow approximately 50% of the beam to pass through. In the bottom panel, it determines the substrate orientation when it is parallel to the incoming beam and sets this orientation as alpha zero. The software is designed to reattempt alignment multiple times if the first scan is unsuccessful.

After data acquisition, optional data processing steps — including data calibration, standard data reduction, data analysis, autonomous decision-making algorithms — could be performed. This lays the groundwork for future intelligent data acquisition and accelerated materials discovery through closed-loop, autonomous experimentation and ML/AI tools.

Once measurement is complete, each bar is removed from the sample holder and helium chamber, then either returned to its original position on the tray or placed in a designated tray for processed samples. This automated handling ensures consistent sample management, minimizes human error, and reduces beamline downtime, resulting in an efficient and reproducible experimental workflow.

This process repeats for each bar until the run is complete.

### 3.4 GIWAXS Sample Alignment and Data Acquisition

Each sample undergoes a standardized alignment protocol optimized for GIWAXS measurements - in LabVIEW (Figure 6). The beam size is approximately 300 μm in height and 700 μm in width, with the x-ray energy fixed at 10 keV. Using a diode in the beam stop that sits in front of the detector (Figure S3), the procedure begins with a vertical (*Y*-axis) scan to position the sample such that approximately 50% of the incident X-ray beam intersects the sample surface. This ensures consistent beam-sample interaction across measurements. Next, an angular (α) scan is performed to determine the orientation at which the substrate surface is parallel to the incident X-ray beam. This orientation is defined as α = 0°, serving as the reference angle for all subsequent measurements. The software is designed to reattempt alignment multiple times if the first scan is unsuccessful. If the automatic alignment still fails for any reason, an error message is recorded, and the system proceeds to the next sample. If multiple consecutive failures occur, beamline staff are notified via email to allow timely intervention. The sample orientation in the direction perpendicular to the incident beam—referred to as phi—is initially set during the robotic GIWAXS setup stage and is not currently adjusted by automated routines, although tools to fine tune phi via edge detection are currently being developed.

Following alignment, data acquisition proceeds at a user-defined incident angle (angles) and exposure time. To accommodate the wide variability in sample morphology—ranging from crystalline to semi-crystalline and from highly ordered to disordered structures—an automated exposure routine is employed. This routine performs a short test exposure (0.1 s) to assess max exposure time allowable without saturating the detector, i.e., ensuring the brightest pixel is below one million counts for Pilatus 2M. If needed, users may also define an upper limit for exposure time (e.g., 5 seconds) to balance beamtime availability, total number of samples to measure, and the risk of radiation-induced damage. The ultimate exposure chosen in this auto exposure mode will be the lesser of the above two. After data collection for a given sample is complete, the motorized sample bar advances to the next predefined position. The alignment and acquisition processes are then automatically repeated, enabling high-throughput GIWAXS measurements with minimal user intervention. To ensure accuracy, the number and positions of samples on each bar are verified immediately prior to measurement using a camera and image recognition system, thereby mitigating potential discrepancies with user-provided metadata.

### 3.5 Control Software

The core components of the system—the helium chamber, sample tracking interface, UR5e robotic arm, and data acquisition—are coordinated through a modular software architecture designed to enable automated, high-throughput GIWAXS measurements. The control software is primarily composed of two applications: the Robot Application and the Robot GUI (Figure 7).

**Figure 7:**
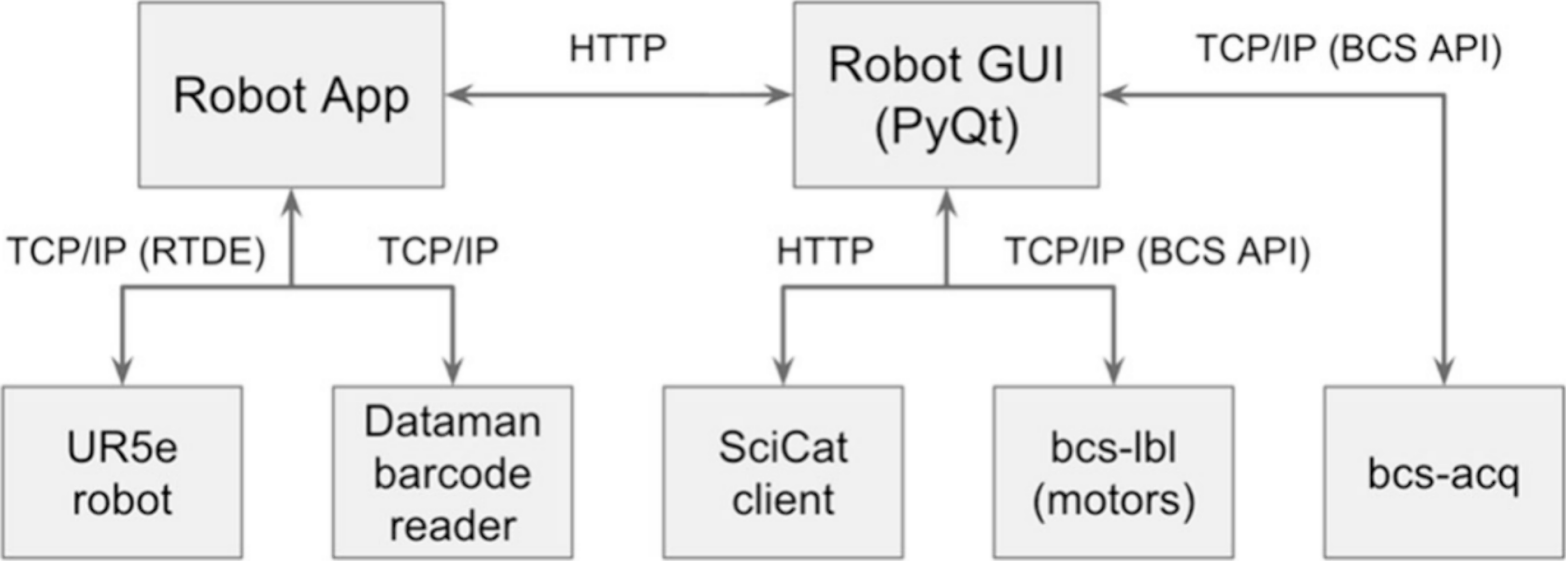
Controls software architecture.

The Robot Application is a Python-based web server that runs on a physical beamline machine via Docker. It communicates with both the UR5e robot and the Dataman barcode reader through a dedicated local Ethernet connection over TCP/IP. This application receives commands from the Robot GUI to execute specific tasks, including ingesting the sample tray and loading or unloading sample bars. Upon receiving a request, the Robot Application instructs the UR5e robot to carry out one of three predefined states: INGEST, LOAD, or UNLOAD. During the INGEST process, the robot picks up each bar from the tray and uses the Dataman barcode reader to scan its QR code. In the LOAD state, it places a selected bar into the sample holder, while in the UNLOAD state, it removes the bar and returns it to its original location on the tray. During both loading and unloading, the robot also automatically controls the helium chamber gate valve to preserve the inert environment.

The Robot GUI is a PyQt-based interface that acts as the main user control panel and links the robotic system to other beamline infrastructure. It connects to the SciCat client to retrieve sample metadata that users have previously entered through the sample tracking interface (see Section 3.2). This metadata includes information such as the physical locations of samples on the bar, relevant incident angles, and specific data acquisition parameters. The GUI also communicates with two beamline control machines: bcs-lbl and bcs-acq. Through the BCS API (SI) using a ZeroMQ (ZMQ) server, the GUI interacts with bcs-lbl to control fine motor movements of the sample stages, including X and Y translation, incident angle (Alpha), and phi. The bcs-acq machine is responsible for controlling the helium purge system, performing GI alignment routines, and acquiring detector images.

### 3.6 Data Storage

Previously, our local data server, with a capacity of 30 TB, was filling up every 1–3 months depending on the nature of the in situ measurements. Emerging capabilities such as high-throughput robotic GIWAXS and time-resolved in situ experiments^25,29,30^ using faster and larger 2D detectors are expected to significantly increase data generation rates. To overcome the limitations of local storage and to support growing computational demands, the National Energy Research Scientific Computing Center (NERSC), a DOE supercomputing facility at LBNL, has been integrated into our experimental data infrastructure.

Facilities such as ALS at LBNL and the Linac Coherent Light Source (LCLS) at SLAC have implemented automated data transfer systems to NERSC. For example, the ALS micro-tomography beamline (8.3.2) utilizes such a system for real-time data processing.^31,32^ These automated data pipelines leverage dedicated Data Transfer Nodes (DTNs) at both ALS and NERSC. Coordination of data transfer and processing is handled through a suite of software tools, including LabVIEW-EPICS, SPLASH, SciCat, and Globus. These tools collectively monitor data acquisition completion, annotate datasets with metadata, initiate file transfers via Globus end points, and verify successful delivery to NERSC. This integrated infrastructure enables scalable, reproducible workflows that support timely data analysis and efficient beamline usage.

Given that NERSC undergoes regular scheduled maintenance—typically monthly—and is occasionally subject to unplanned outages, maintaining synchronized copies of newly acquired data at both local and remote (NERSC) locations is a practical and resilient strategy. This ensures continuous data accessibility for users during beam time and in the critical weeks following, when data analysis activity is typically at its peak.

## 4 Current Developments and Future Directions

The work presented here establishes a high-throughput, intelligent framework for the efficient and reliable characterization of pre-fabricated thin-film samples. A real-time data reduction and analysis pipeline is currently under development (manuscript in preparation), capable of performing key tasks such as data calibration, conversion of raw detector images to 1D line profiles, and peak fitting. Smart algorithms, including gpCAM, will be used to synthesize peak-fitting results—such as peak positions and widths—alongside sample composition information in real time. This will enable the system to selectively measure only a representative subset of pre-fabricated samples while still meeting beam time objectives, thereby optimizing photon usage and experimental efficiency.

One frequently reported bottleneck by users is the premature depletion of prepared samples during limited beam time, which undermines experimental productivity and slows progress in high-throughput materials discovery. To address this challenge and further streamline synchrotron workflows, we have procured a robotic sample processing platform from Sciprios GmbH (Germany). This system automates thin-film fabrication through solution mixing, spin coating, and thermal annealing.

Initially, this robotic processor will be deployed offline in a dedicated ALS chemical laboratory. While it does not fabricate samples directly at the beamline, it enables more rapid and reproducible production of thin films that can then be manually loaded into a sample garage or mounted on the measurement stage. This is especially advantageous for protocols with short processing times - its utility is limited for samples requiring slow or prolonged processing steps, such as multi-hour thermal annealing or overnight vacuum drying.

By integrating this processing platform into the broader measurement workflow, we aim to enable autonomous, closed-loop experimentation in which thin films are fabricated directly at the beamline and characterized in real time based on ongoing experimental results. This capability will facilitate on-the-fly sample generation and adaptive measurement strategies, both of which are critical for intelligent materials discovery pipelines.

Looking ahead, we plan to incorporate pre-characterization tools—such as optical microscopy, UV-vis spectroscopy, Raman and photoluminescence (PL) measurements—into the robotic processing platform. These tools would enable early assessment of sample quality prior to beamline measurement, allowing the system to make data-informed decisions about which samples to prioritize, thereby enhancing both efficiency and scientific output.

As part of the ALS Upgrade, the SAXS/WAXS beamline will be relocated to Beamline 4.3.1, which is expected to provide approximately an order of magnitude higher flux. The advanced optics at BL 4.3.1 will provide multiple beam sizes and shapes, including a line-shaped beam that better matches typical sample dimensions in GI geometry, thereby improving photon utilization by an additional factor of 5–10. Together with ongoing advances in robotics, high-performance computing resources such as Doudna at NERSC, and autonomous experimentation capabilities, these upgrades are expected to significantly increase experimental throughput, enhance measurement speed, and accelerate materials discovery.

## Supplementary Material



## Notes

Additional details on the LabView interface for monitoring oxygen levels and helium gas flow, the Kapton blocker with corresponding GIWAXS data (blocker in/out), as well as the sample tracking interface, sample tray, beam size, sample size, bar dimensions, and vendor information (PDF)
