# Supplementary material for: High-Throughput Robotic GIWAXS at ALS SAXS/WAXS Beamline
Source: Photon Sci. 2025 Oct 22;1(1):35–44. doi: 10.1021/photonsci.5c00017 (PMC12884978; doi:10.1021/photonsci.5c00017)
Supplement: Supplementary file 1 [file po5c00017_si_001.pdf]

Supporting Information

High Throughput Robotic GIWAXS at ALS SAXS/WAXS beamline

Eric Schaible[a], Ivan Galikeev[a], Matthew Roizin-Prior[a], Garret Birkel[a], Yunfei Wang[a,b], Wiebke Koepp[a], Raja Vyshnavi Sriramoju[a], Harold Barnard[a], Chinweike Osubor[a], Camille Molsick-Gibson[a], Piotr Gach[a], Sujoy Roy[a], Xiaodan Gu[b], Dylan McReynolds[a], Alexander Hexemer[a], Lucas Kistulentz[a], Damon English[a], Dilworth Y Parkinson[a*], Chenhui Zhu[a*]

a. Advanced Light Source, Lawrence Berkeley National Laboratory, Berkeley, CA 94720, USA

b. School of Polymer Science and Engineering, University of Southern Mississippi, Hattiesburg, MS 39406, USA

***chenhuizhu@lbl.gov; dyparkinson@lbl.gov**

Experimental Section

This development was carried out at the ALS SAXS/WAXS beamline (7.3.3). The beam size was approximately 300 µm (height) × 700 µm (width), with the X-ray energy fixed at 10 keV. At low incident angles (e.g., 0.2°), the tall vertical profile produces an elongated footprint of ~8 cm (beam height / sin 0.2°), which far exceeds the typical 1 cm sample length along the beam direction. As a result, a substantial fraction of photons do not interact with the sample. To mitigate this limitation, the new SAXS/WAXS beamline (4.3.1) planned for the ALS upgrade will incorporate optics that support multiple modes, including one providing a line-shaped beam with a reduced vertical size of ~50 µm, thereby improving photon utilization for GIWAXS/GISAXS.

At present, samples are typically 12 mm long (along the beam direction) and 10 mm wide. The 10 mm width is not strictly necessary and may be reduced (e.g., to 5 mm), allowing a greater number of samples per bar. Conversely, users may prepare wider samples if desired, although this reduces measurement throughput by decreasing the number of samples accommodated per bar per load.

Off-the-shelf aluminum sample bars (½″ × ½″ × 8″) can be purchased from vendors such as McMaster-Carr (part #9447T36) or fabricated at users' home institutions.

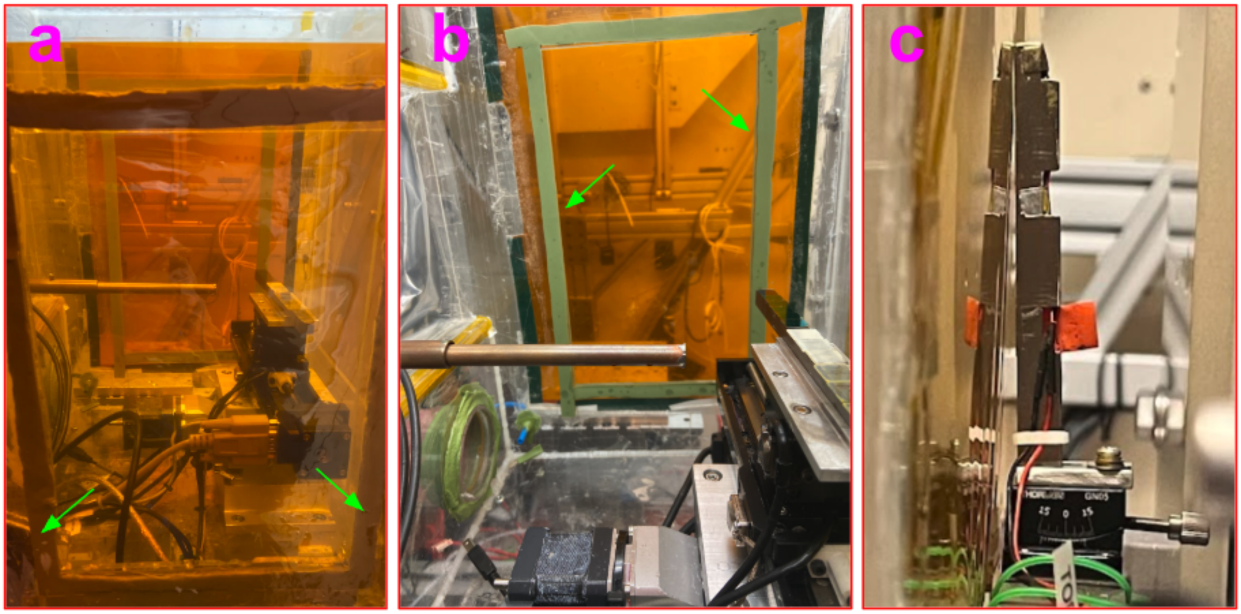

Figure S1 In the previous setup: (a) exterior view of the helium box, (b) interior view, and (c) beam stop assembly positioned immediately downstream of the Kapton exit window. The beam stop served two purposes: (1) to block background scattering from the Kapton caused by the transmitted beam, and (2) to measure the transmitted beam intensity downstream of the samples using a photodiode mounted at the top of the beam stop. Green arrows indicate the sealed edges of the window for manual sample loading and unloading.

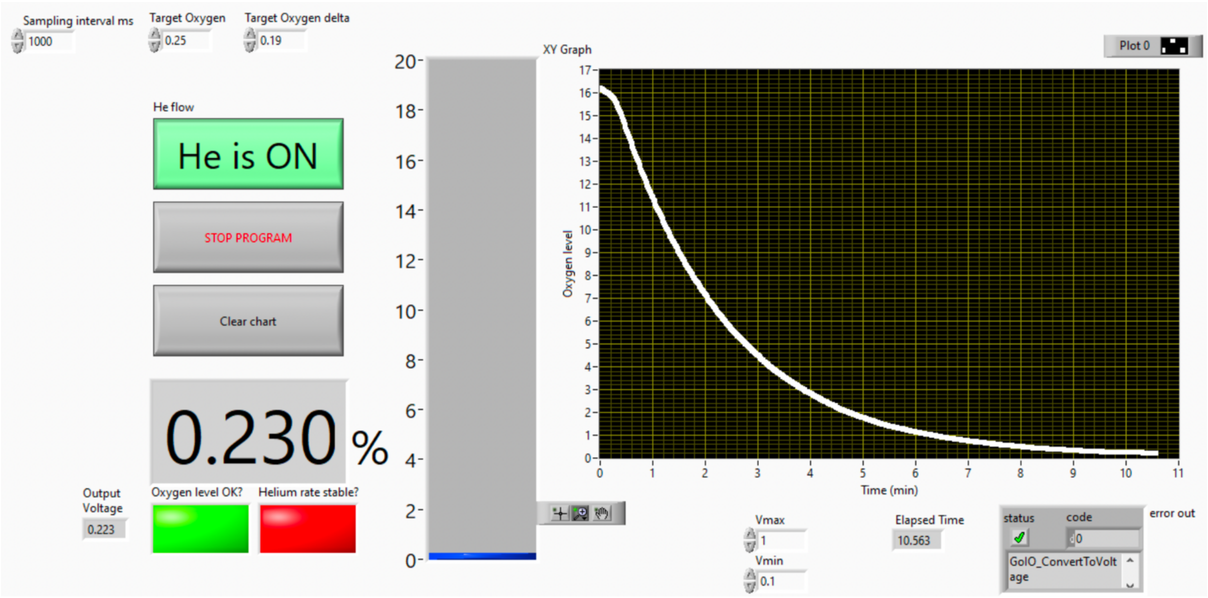

Figure S2 The LabVIEW interface for oxygen level and helium gas flow control. The interface allows users to switch helium flow on or off, define maximum and minimum flow rates, and specify a target oxygen concentration. A real-time plot on the right displays oxygen concentration (%) as a function of time (minutes), showing that the oxygen level decreases below 0.25% within 10 minutes.

Improvements in the new helium chamber design

The new chamber design was developed to provide a better seal with fewer potential leakage points. In the previous setup, the upstream entrance of the box was sealed by wrapping a plastic bag around the end of the synchrotron beam pipe—a laborious and unreliable method. The new design also accommodates higher helium flow rates. In the earlier configuration, the beam stop was positioned immediately outside the box, in contact with the exit Kapton window (Fig. S1c). At elevated helium flow rates, the Kapton window could bulge and press against the beam stop, potentially displacing the diode within the beam stop and interrupting GI alignment. The new design (with the Kapton blocker inside the chamber, Fig S3) eliminates this issue, yielding a more robust system capable of sustaining higher helium flow during the initial purging process.

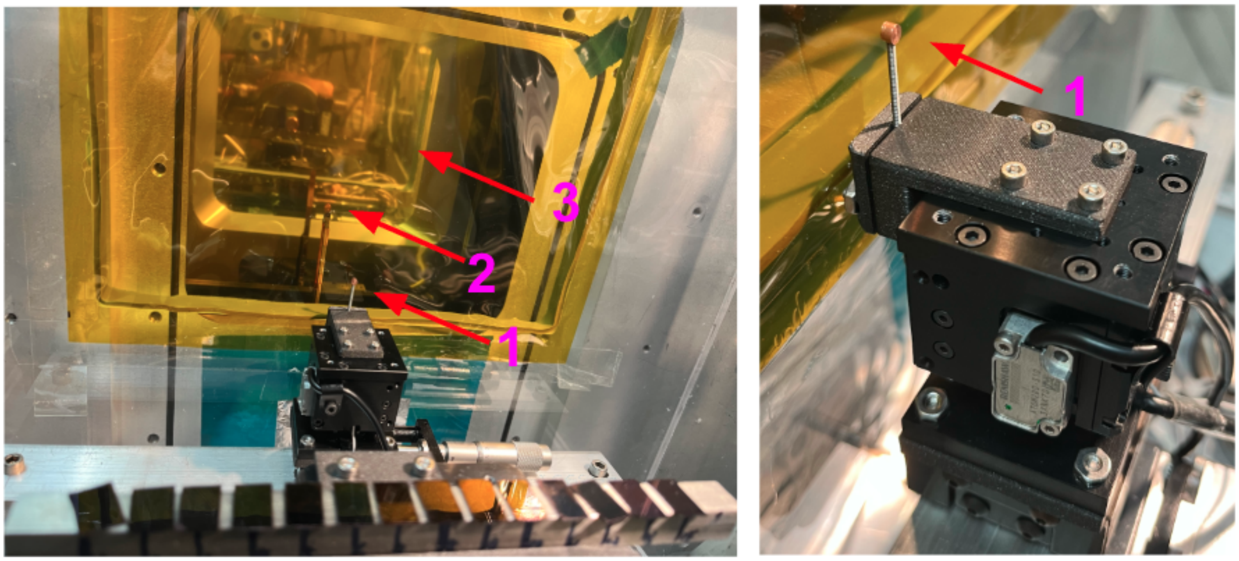

Figure S3 The new beam stop assembly consists of two components: (1) a Kapton blocker and (2) a secondary beam stop housing a photodiode, positioned directly in front of the Pilatus 2M detector (3).

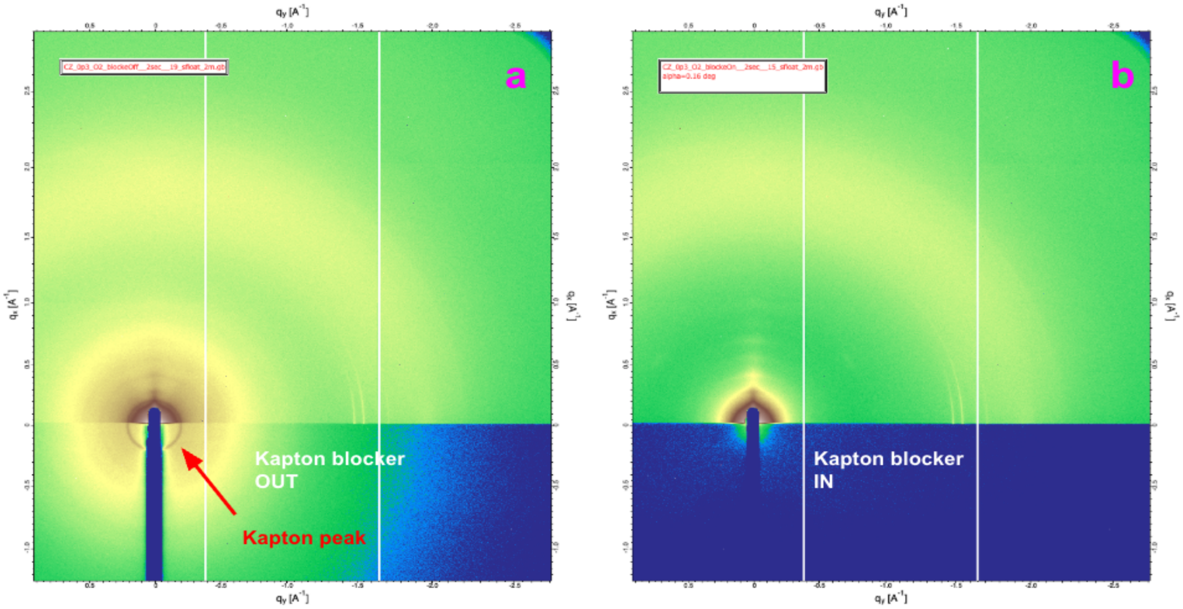

Figure S4 GIWAXS pattern (raw detector images) of a weak-scattering sample, with the Kapton blocker out and in.

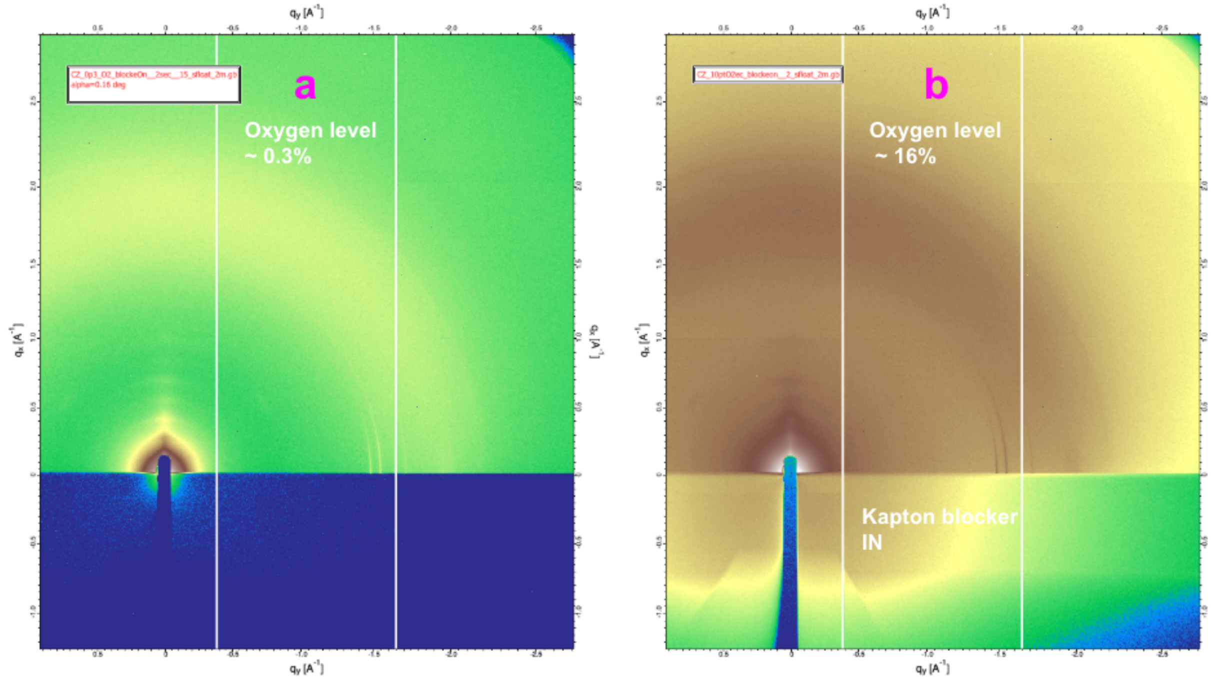

Figure S5 GIWAXS patterns (raw detector images) of a weakly scattering sample collected at oxygen levels of ~0.3% (left) and ~16% (right), demonstrating that the helium environment substantially improves data quality.

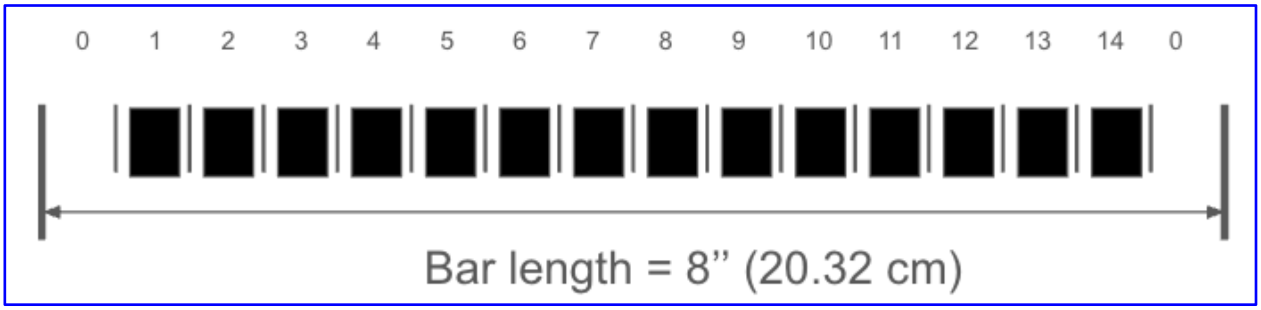

Figure S6 Illustration of sample spacing on the bar. The far-left and far-right positions are reserved to facilitate bar handling during packing, shipping, and loading/unloading.

Sample tracking interface

The sample tracking application is written in TypeScript, using the React library. It’s served using Nginx running in a virtual machine. It stores data in an instance of the SciCat project, deployed specifically for the sample tracker. The sample tracker and the SciCat instance are deployed to Google Kubernetes Engine, with additional configuration to allow communication with a user metadata service that is hosted at LBNL. Helm is used to reduce the complexity of managing the Kubernetes manifests that define and connect these services.

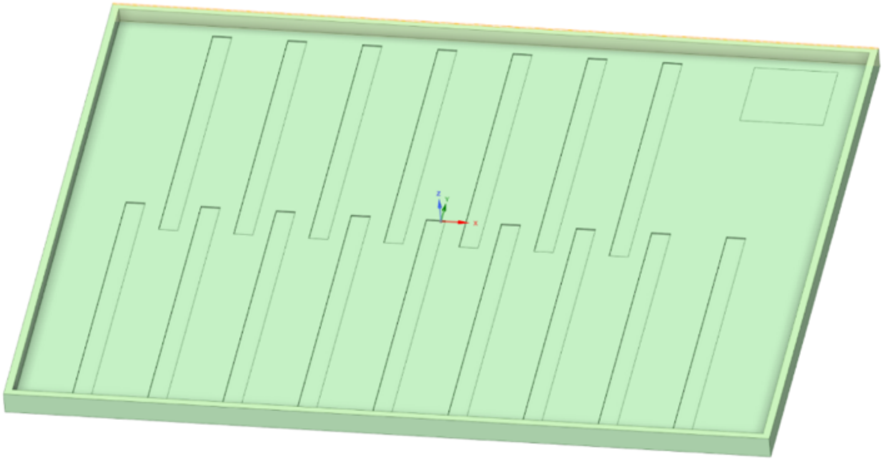

Figure S7 The current sample tray (bar garage) design accommodates sixteen bars, each capable of holding fourteen 1 cm-wide samples. The total capacity can be increased by 50% or more by reducing the sample width, if required. Under typical operating conditions—including helium gas flow overhead, GI alignment, measurements at multiple spots and incident angles, and tiled image collection (two detector images acquired at slightly different vertical positions, e.g., 5 mm, and subsequently stitched to eliminate horizontal detector module gaps)—processing a full tray of bars is estimated to require approximately twelve hours or longer.

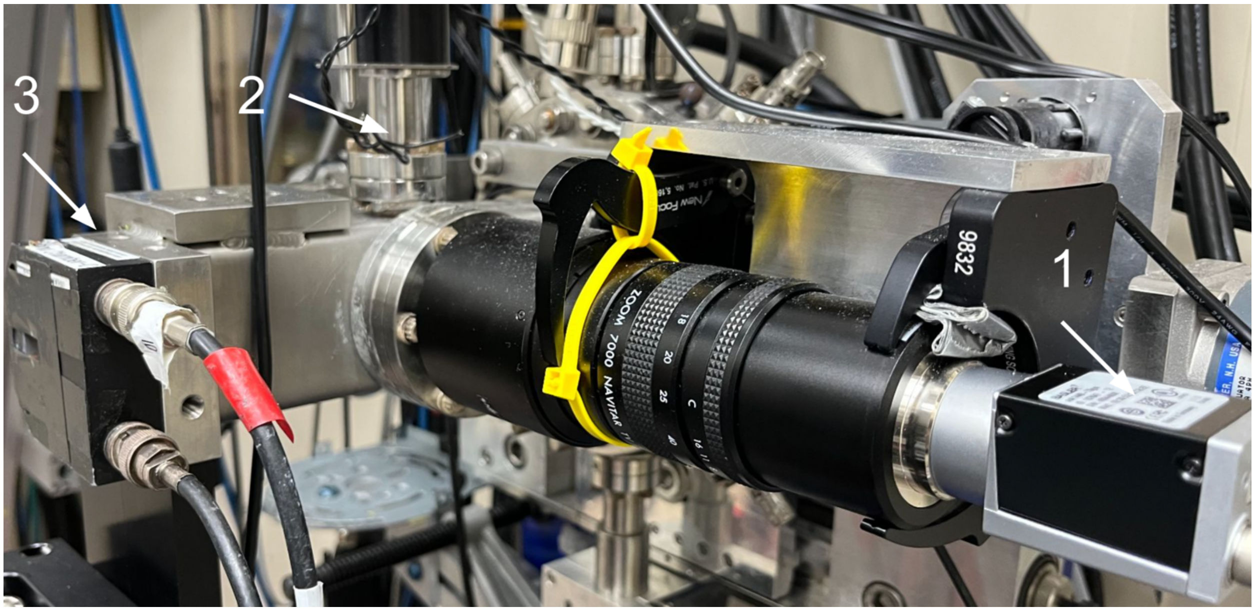

Figure S8 CCD camera setup for monitoring the beam position relative to the pinhole within the pinhole assembly. (1) CCD camera with optical lens assembly; (2) device for adjusting the mirror position inside the beam pipe; (3) end of the synchrotron beam pipe with an ion chamber (I_0) for measuring the incoming beam intensity. The mirror is inserted into the beam pipe approximately 10 cm upstream from its end.

BCS API: More information can be made available upon request.
